# Effect of HO-1-modified BMMSCs on immune function in liver transplantation

**DOI:** 10.1038/s41598-022-06141-7

**Published:** 2022-02-23

**Authors:** Peng Li, Yuyi Zhang, Qiongxia Li, Yubo Zhang

**Affiliations:** 1 Department of Hepatobiliary Surgery, Xiangyang No. 1 People’s Hospital, Hubei University of Medicine, Xiangyang, 441000 China; 2 Department of Obstetrics and Gynaecology, Xiangyang No. 1 People’s Hospital, Hubei University of Medicine, Xiangyang, 441000 China; 3 Department of Digestive Endoscopy Centre, Xiangyang No. 1 People’s Hospital, Hubei University of Medicine, Xiangyang, 441000 China; 4 Department of Stomatology, Xinchang Hospital Affiliated with Wenzhou Medical University, Shaoxing, 312500 China

**Keywords:** Immunology, Stem cells

## Abstract

We examined whether haem oxygenase-1 (HO-1) could enhance the immunosuppressive effects of bone marrow mesenchymal stem cells (BMMSCs) on the rejection of transplanted liver allografts in rats. The animals were divided into three groups: the normal saline (NS) group, BMMSC group and HO-1/BMMSCs group. In vitro, the extraction, culture and HO-1 transfection of BMMSCs were performed. Mixed lymphocyte response (MLR) analysis of HO-1/BMMSCs efficacy was performed. The rejection model of orthotopic liver transplantation in rats was established when BMMSCs and HO-1/BMMSCs were transfused via the portal vein. To reduce research bias, we established an isogenic Liver transplantation model of (LEW → LEW) and (BN → BN), which can achieve tolerance. Changes in histopathology and liver function in the transplanted liver and changes in regulatory T cell (Tregs), natural killer (NK) cells and cytokines after transplantation were observed in the different groups. The severe acute rejection after liver transplantation on postoperative Day 10 was observed in the NS group. The BMMSC group showed strong protective effects against rejection within the first 10 days after transplantation, while HO-1/BMMSCs showed stronger effects on rejection than BMMSCs alone. In addition, the activity of natural killer (NK) cells decreased significantly, the levels of regulatory T cells (Tregs), interleukin-10 (IL-10) and transforming growth factor-β (TGF-β) increased significantly and the levels of interleukin-2 (IL-2), interleukin-6 (IL-6), interleukin-17 (IL-17), interleukin-23 (IL-23), tumour necrosis factor-α (TNF-α) and interferon-γ (IFN-γ) decreased significantly in the HO-1/BMMSC group compared with the BMMSC group. HO-1/BMMSCs showed better immunosuppressive effects after liver transplantation than the other treatments. Our findings reveal that HO-1 can enhance the effects of BMMSCs on inhibiting acute rejection in orthotopic liver transplantation in rats.

## Introduction

Liver transplantation (LT) is an effective treatment for patients with liver failure and end-stage liver disease. However, the rejection of liver transplantation is obvious. The existing immunosuppressive treatments are effective but bring some side effects, such as malignant tumours and infections, which affect the long-term survival rates of liver transplant recipients and restrict LT activity.

Immune tolerance is a long-term, nonresponsive state of the recipient's immune system to the allograft and a normal immune response to other foreign antigens. Therefore, how to induce durable and drug-free immune tolerance is an urgent problem to be solved^1^.

Bone marrow mesenchymal stem cells (BMMSCs) have important application value in organ transplantation^2^. BMMSC have low immunogenicity and a certain effect on inhibiting T cell-mediated immune rejection after organ transplantation^3,4^. These cells can secrete some immunosuppressive cytokines, such as interleukin-10 (IL-10) and transforming growth factor-β (TGF-β), and interfere with helper T lymphocyte differentiation to induce immune tolerance^5,6^. This is a promising treatment strategy. However, it has also been reported that the effect of BMMSC infusion alone on tissues is low, and studies have shown that genetic engineering of BMMSC is a more effective method^7^. HO-1 is an immunomodulatory factor involved in the regulation of immune tolerance after organ transplantation^8^. HO-1 can be transfected into BMMSC to enhance their immunomodulatory and antioxidant capacity while also prolonging their action time^9^.

BMMSC and HO-1 are immunoprotective in liver transplantation. Therefore, based on the findings of previous studies, we hypothesized that HO-1 could cooperate with BMMSCs to play a protective role in grafts. In this study, the rat HO-1 gene was transfected into BMMSC to form HO-1/BMMSCs in vitro and subsequently study its effect on orthotopic rat liver transplantation. We examined whether HO-1 could enhance the regulatory effect of BMMSC on transplant immunity and its potential mechanism.

## Results

### Culture and identification of BN rat BMMSCs

**Figure 1:**
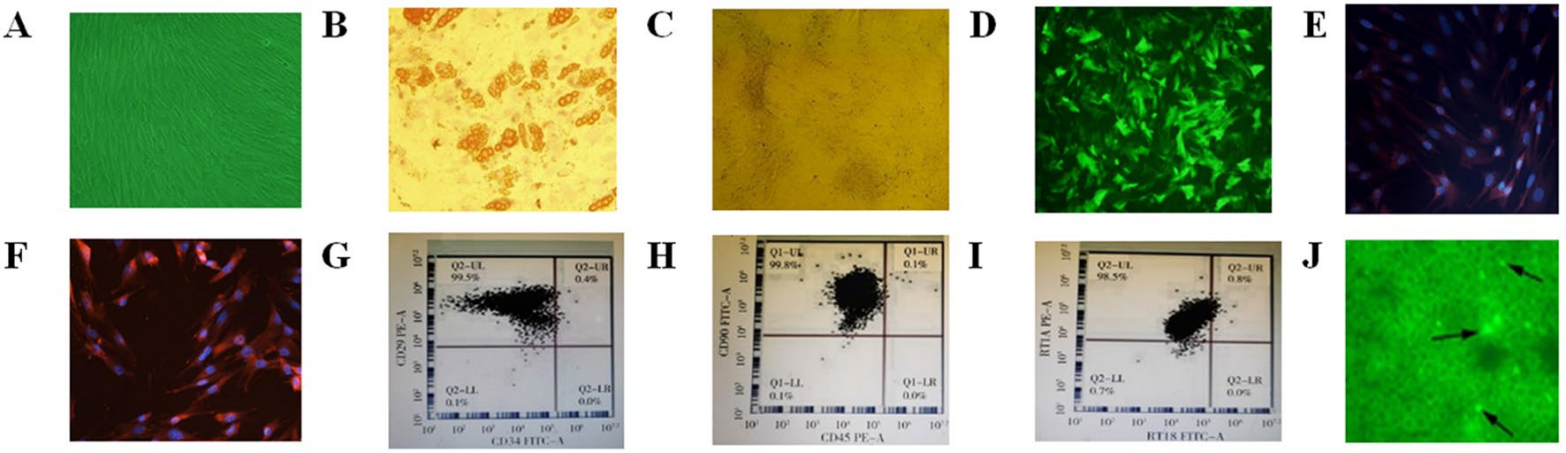
Morphology, characteristics, phenotype and HO-1 transfection of BMMSCs. (**A**) The morphology of BMMSCs in the third generation (×100). (**B**) Adipogenic analysis of BMMSCs. (**C**) Osteogenic verification of BMMSCs (×200). (**D**) Dark field image of BMMSCs transfected with HO-1 (×100). (**E**) HO-1 expression in BMMSCs without HO-1 transfection. (**F**) HO-1 expression in BMMSCs transfected with HO-1. The expression level was significantly higher than that in the untransfected group (×200).The phenotype of BMMSCs was examined. (**G**) The positive rate of CD29^+^CD34^+^ cells. (**H**) CD90^+^CD45^+^ cells were 99.8%, (**I**) The positive rate of RT1A^+^RT1B^−^ was 98.5% (Red in **E**). (**F**) indicates HO-1 protein staining, Blue indicates nuclear staining. (**J**) The black arrow indicates HO-1/BMMSCs in the transplanted liver.

Rat BMMSCs were successfully cultured and expanded in vitro. ① The cells adherently grew. The cells after being passaged were long, fusiform and arranged in a vortex or chrysanthemum shape with typical BMMSCs morphological features (Fig. 1A). ② The cells could differentiate into adipocytes (oil red O staining showing red lipid droplets in the cytoplasm, Fig. 1B) and osteoblasts (von Kossa staining showing black calcium in cells) (Fig. 1C). ③ Analysis of cell surface markers showed the expression of CD29^+^CD34^−^, CD90^+^CD45^−^ and RT1A^+^RT1B^−^ in 99.5%,99.8% and 98.5% of cells, respectively (Fig. 1G–I).

### Recombinant adenoviral vector-mediated transfer of the HO-1 gene to BMMSCs and its efficacy

BMMSCs were infected with recombinant adenovirus expressing HO-1, and the GFP gene was used as a reference. The cells were observed 48 h after infection. GFP expression in the cells was > 80% by fluorescence microscopy (Fig. 1D). Ad-HO-1 was identified by immunocytochemical staining. Red-labelled HO-1 expression in the HO-1/BMMSC group (Fig. 1F) was significantly higher than that in the BMMSC group (Fig. 1E). The expression of the HO-1 gene was upregulated, and the expression level was approximately 5 times that of the control group. HO-1/BMMSCs accumulated in the transplanted liver until 24 days after surgery (Fig. 1J). These results indicate that adenovirus-mediated HO-1 was successfully expressed in BMMSCs and was the foundation for the next experiment.

### The mixed lymphocyte reaction (MLR) results

**Figure 2:**
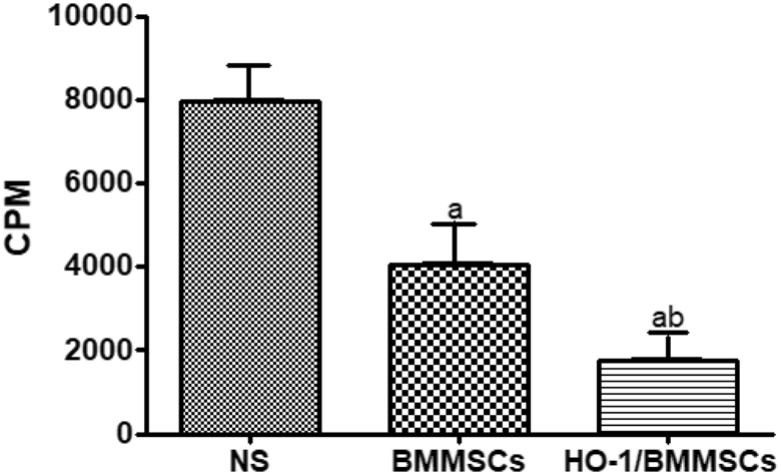
Differences in MLR between recipient treatment groups. The HO-1/BMMSC group compared with the BMMSC group (^b^P < 0.05). The BMMSC group compared with the NS group (^a^P < 0.05). The HO-1/BMMSC group compared with the NS group (^ab^P < 0.05).

In the MLR experiment, we found that the BMMSC group had significantly inhibited lymphocyte proliferation, and the inhibition rate was as high as 49.37%, while in the HO-1/BMMSC group, the inhibition rate was as high as 77.22%, showing a stronger inhibitory effect (Fig. 2). Compared with the NS group, there was a significant difference (P < 0.05).

### A liver transplantation rejection model (BN → LEW) was successfully established

In the NS group, during the first two days after surgery, spirit, diet, drinking and activity were normal. From the fifth day after surgery, 15 rats began to exhibit reduced intake, weight loss, reduced mental condition, and gradually darker urine colour. Alertness to stimulation in the surrounding environment decreased obviously. On the 7th day, most rats experienced spiritual malaise, significant weight loss, messy hair, and darker urine. On the 10th day, the animals were unwilling to move by themselves. They were insensitive to environmental stimuli and conspicuously emasculated. Their urine was dark yellow. Their fur began to fall off, and most of the animals died. In the BMMSC group, mental status and appetite remained normal until the 10th day, and body weight gradually increased. From the 10th day, 11 rats began to exhibit reduced intake, weight loss, reduced mental conditions, and gradually darker urine colour. Alertness to stimuli in the surrounding environment decreased obviously. By the 15th day, most of the rats died. In brief, the acute rejection symptoms of the recipients were reduced to some extent, and the mental state, activity, survival rate and response to external stimuli were improved, but the overall effect was still not very satisfactory. The observations in the HO-1/BMMSC group were very encouraging and most of the recipients survived well without typical symptoms of acute rejection. The mental status and appetite of rats in the HO/BMMSC group remained normal until 20 days after surgery, and their body weight gradually increased. On the 20th day, a few rats began to exhibit reduced intake, weight loss, reduced mental conditions, and a darker urine colour. Alertness to stimuli in the surrounding environment decreased obviously. By the 25th day, most of the rats died. These results are shown in Table 1. During the entire observation period, the median survival time was 10 days in the NS group, 15 days in the BMMSC group and 24 days in the HO-1/BMMSC group. HO-1/BMMSC treatment further improved the survival rates of transplanted rats. The difference was statistically significant compared with that in the BMMSC group.

**Table 1:** Clinical evaluation after LT.

Treatment	0 d	1 d	5 d	7 d	10 d
NS	N	N	N^**-**^	R	R^**+**^
BMMSCs	N	N	N	N	N^**-**^
HO-1/BMMSCs	N	N	N	N	N

N = No rejection, R = Rejection, R^+^ = Aggravation of rejection, N^−^ in the NS group = 15 rats showed some signs of rejection. N^−^ in the BMMSC group = 11 rats showed some signs of rejection.

### Pathological manifestations and liver function of the transplanted livers in each group

**Figure 3:**
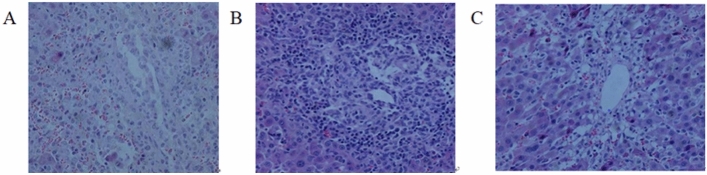
Pathological findings of LT in each group (×100). (**A**) NS Group. (**B**) BMMSC group. (**C**) HO-1/BMMSC Group.

According to the Banff criteria, the transplanted liver in the NS Group 10 days after the operation showed severe rejection with mixed lymphocyte infiltration in the portal vein area, inflammation and destruction in interlobular bile duct, inflammatory cell infiltration under the interlobular vein and central vein endothelium, and hepatocyte necrosis (Fig. 3A). In the BMMSC group, only mild rejection was observed. Pathological examination showed that a small amount of lymphocyte infiltration occurred in the portal area. Bile duct epithelial cell degeneration and cholangitis were rarely observed. Interlobular phlebitis was not common (Fig. 3B). Compared with that in the other two groups, there was no obvious immune rejection or pathological damage in the HO-1/BMMSC group, which was close to normal (Fig. 3C). The liver function of the recipients in the HO-1/BMMSC group was significantly better than that in the NS group and BMMSC group. The difference was statistically significant (Table 2).

**Table 2:** The liver function of the recipients at 10 days after liver transplantation (mean ± SD, n = 5).

Treatment	TBiL (μmol/L)	ALT (IU/L)	AST (IU/L)
NS	187.6 ± 38.6	393.2 ± 33.9	361.8 ± 59.6
BMMSCs	51.7 ± 6.2^a^	135.4 ± 8.5^a^	113.4 ± 11.6^a^
HO-1/BMMSCs	13.6 ± 4.3^ab^	28.2 ± 4.5^ab^	20.3 ± 7.5^ab^

^a^BMMSC group compared with NS group (P < 0.05). ^b^HO-1/BMMSC group compared with BMMSC group(P < 0.05). ^ab^HO-1/BMMSC group compared with control group (P < 0.01).

### HO-1 overexpression in BMMSCs induces persistent high levels of splenic Tregs in rats

**Figure 4:**
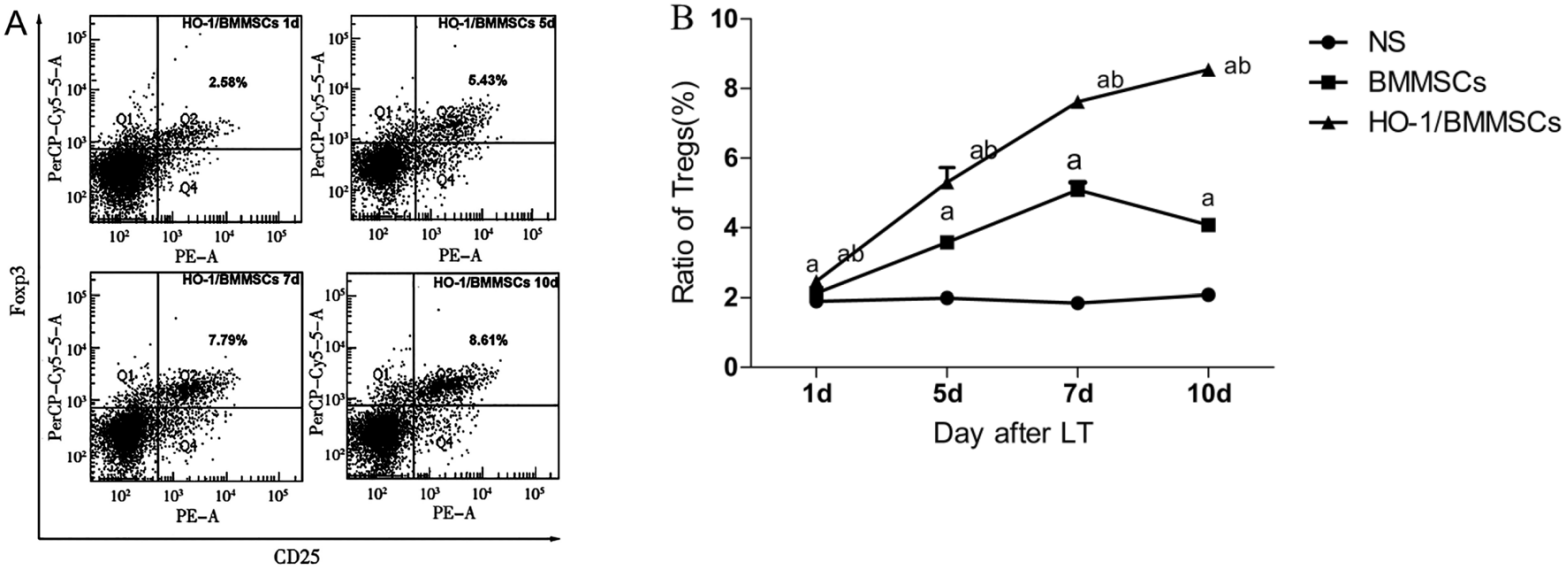
The level of Tregs in recipient spleens. (**A**) Scatter plot showing Tregs in the spleen in the HO-1/BMMSC group at different time points after the operation. (**B**) Treg ratios of rats in each group (compared with the NS group, P < 0.05; compared with BMMSC group, P < 0.05).

Flow cytometry showed that the HO-1/BMMSC group had significantly increased the levels of CD4^+^CD25^+^Foxp3^+^ Tregs, which peaked at the 7th day and fell at the 10th day (Fig. 4A). The Treg ratios in the BMMSC and NS groups are presented in Fig. 4B. The Treg levels in the HO-1/BMMSC group showed a rapid and steady increase and remained high on the 10th day. Statistical analysis showed that the HO-1/BMMSC group had further increased Treg levels compared with the BMMSC group, especially on the 10th day. The difference was statistically significant (Fig. 4B).

### Overexpression of HO-1 enhances the production of cytokines in rat serum by BMMSCs (Fig. 5)

**Figure 5:**
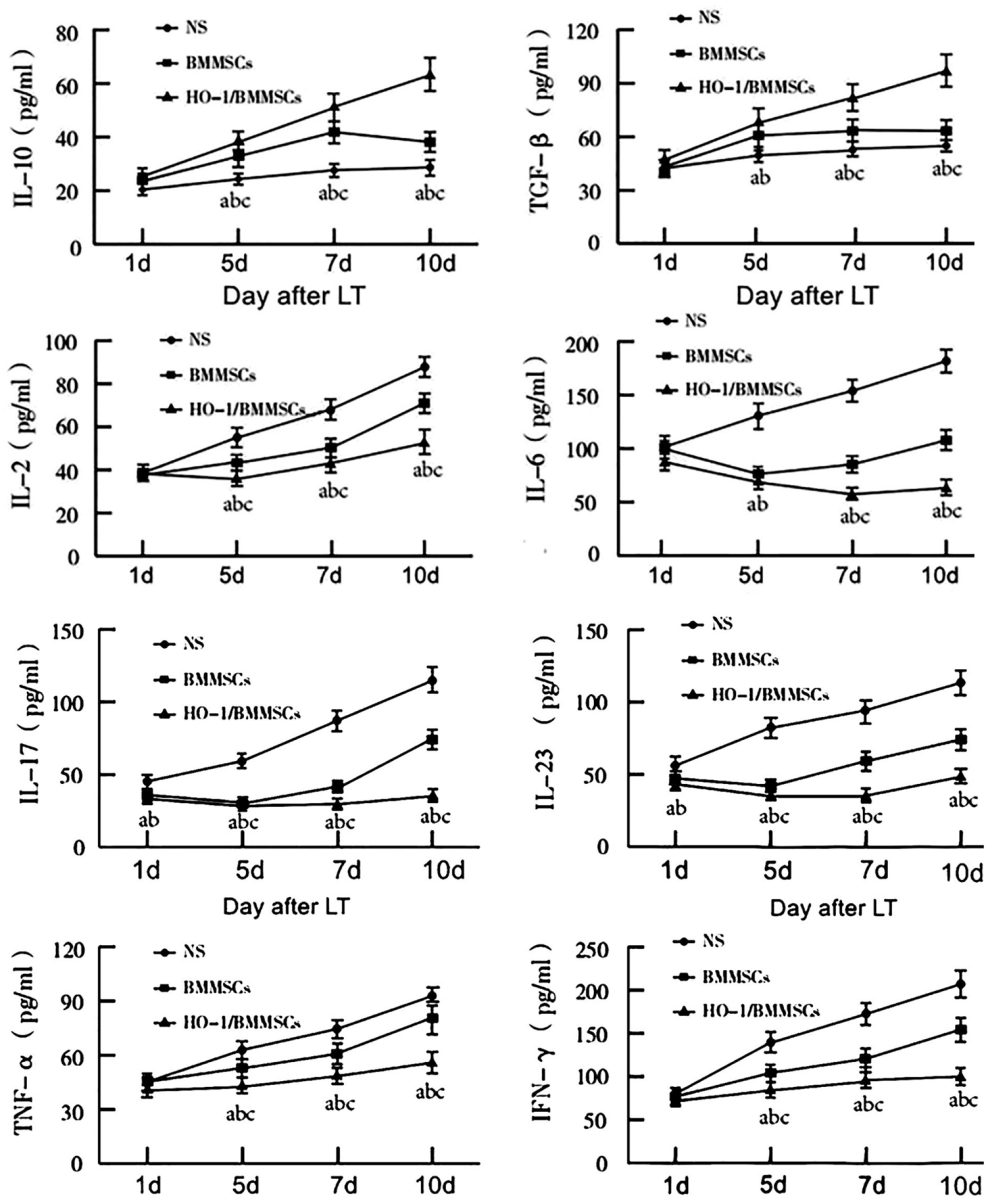
Serum levels of various cytokines. ^a^P < 0.05, compared with the NS group. ^b^P < 0.05, compared with the BMMSC group. ^c^P < 0.05, compared with the HO-1/BMMSC group.

The ELISA results showed that the expression levels of cytokines related to anti-inflammatory effects or Treg differentiation, such as IL-10 and TGF-β, were further increased in the HO-1/BMMSC group compared with the NS group and BMMSC group. The increases in IL-2, TNF-α and IFN-γ related to proinflammatory or Th17 cell differentiation were significantly smaller than those in the BMMSC and NS groups. IL-6, IL-17 and IL-23 levels in the HO-1/BMMSC group showed a decrease or small fluctuations but did not decrease first and then increase, as they did in the BMMSC group, and the total expression levels of IL-6, IL-17 and IL-23 were significantly lower than those in the BMMSC and NS groups. The difference was statistically significant.

### Overexpression of HO-1 enhanced the inhibition of NK cell activity in BMMSCs (Fig. 6)

**Figure 6:**
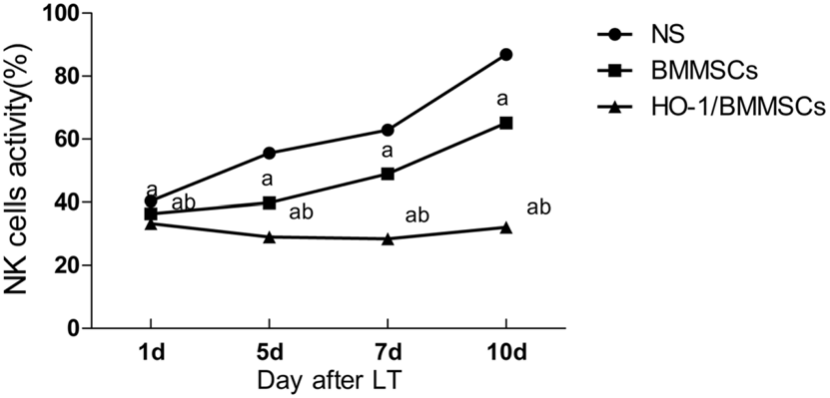
NK cells activity. ^a^P < 0.05, compared with the NS group. ^b^P < 0.05, compared with the BMMSC group.

Although BMMSC treatment significantly inhibited NK cell activity in the BMMSC group, NK cell activity still showed a significant increase at 7 and 10 days. The activity of NK cells in the HO-1/BMMSC group did not significantly increase during the observation period but only fluctuated around normal levels. Statistical analysis showed that NK cell activity was further reduced to near normal levels in the HO-1/BMMSC group, and the difference was statistically significant (Fig. 6).

### Liver transplantation rejection in the syngraft model was not established (LEW → LEW,BN → BN)

To reduce research bias, we established models of (LEW → LEW) and (BN → BN) in 20 rats (10 pairs). The mental status, activity, diet and water intake of each group were very good, with no weight loss and normal liver function. The average survival times were more than 120 days. These results indicated that the syngraft model had no obvious rejection and that immune tolerance was achieved. Therefore, the positive effect of HO-1/BMMSCs was reflected in the allogeneic LT from the syngraft model.

## Discussion

Liver is the organ of preferential immune caused by its special anatomy and immunological characteristics compared with other solid organ transplantation. Clinical liver transplant rejection has been well controlled despite the development of new immunosuppressants, but the incidence of rejection is still in existence. The 1-year, 3-year and 5-year graft survival rates after orthotopic liver transplant are 89.6%, 80.8% and 72.8%, while the 1-year, 3-year and 5-year patient survival rates are 91.8%, 83.8% and 76.1%, respectively^10^. The long-term use of immunosuppressive agents increases the risk of adverse reactions such as hypertension, nephrotoxicity, neurotoxicity, postoperative tumours, and metabolic diseases^11^. Surgical factors in LT such as liver perfusion, resection or vascular anastomosis affect the postoperative survival rate.We established a standardized surgical process through previous continuous training in the model of BN → LEW,LEW → BN,BN → BN,LEW → LEW. We found that isotransplantation has no rejection but allotransplantation occurred rejection approximately 1 week after surgery. BN → LEW has more severe rejection and was more suitable model for research. Therefore, it is necessary to find a new immunosuppressive strategy with few adverse reactions.

Because of their low immunogenicity, low antigen-presenting ability and unique immunomodulatory effects, mesenchymal stem cell(MSC) can affect the immune system, such as T cells, B cells, NK cells, monocytes and dendritic cells, in vivo and in vitro^12–14^, thereby evading immune recognition and suppressing immune responses^15^. At present, MSC have been used in almost all basic research of organ transplantation, and MSC transplanted in vivo have homing characteristics. Under the effect of various factors, exogenous or autologous MSC can directly migrate and colonize targeted tissues^16,17^. MSC have also been shown to play an important role in reducing transplant immune tolerance in organ transplantation^18^.

MSC can migrate to the inflamed colon and exert anti-inflammatory and immunoregulatory effects^19^, but their intestinal colonization rate is extremely low, and MSC are considered to be at least partially therapeutic by paracrine mechanisms. Furthermore, due to ischaemia, hypoxia and inflammatory reactions in local tissues, the survival rate of MSC after transplantation is extremely low, and the number of surviving cells at 4 days after transplantation is significantly decreased, while less than 1% of cells survive more than 1 week^20^. Regardless of the way MSC exert their reparative, anti-inflammatory, and immunomodulatory effects, their effects are severely limited by low survival in vivo. In the BMMSC treatment group, pathological examination at 10 days showed that the tissue structure was severely damaged, a large number of inflammatory cells had infiltrated and apoptotic cells were increased significantly, indicating moderate to severe rejection. Although the rejection extent was lower than that in the NS group, the survival and pathological changes of the recipient rats were significantly worse than before. The various test results suggested that the BMMSC group did not maintain good inhibition of NK cell activity, regulation of cytokine expression levels and the induction of Treg production within 1 week. HO-1 is an inducible form of HO, a cytoprotective enzyme that protects cells by inhibiting inflammatory, antiapoptotic, and oxidative stress effect and ischaemia/reperfusion injury^21,22^. HO-1/BMMSC improved allogeneic LT outcomes by attenuating inflammatory responses and acute cellular rejection, as well as enhancing immunomodulatory effects, compared with BMMSC. However, the mechanism by which HO-1 helps BMMSC attenuate the inflammatory response and acute rejection is unclear^23^. Our research showed that HO-1 also has protective and reparative effects on intestinal damage due to various causes, the main mechanisms of which include antioxidation, anti-inflammatory, antiapoptosis and the regulation of microcirculation^24,25^. In addition, HO-1 has a regulatory effect that can attenuate MSC apoptosis under hypoxia and oxidative stress^26^. However, in organ transplantation, HO-1 also reduces rejection, prolongs graft survival times and even induces graft immune tolerance^27,28^. In this study, BMMSC were genetically engineered to express HO-1. HO-1 transfection can improve the transformation and antioxidant capacity of BMMSC to improve the survival and proliferation ability of BMMSC. On the one hand, it was thought that the cytoprotective enzyme could improve the survival rate of BMMSC in liver-transplanted rats. On the other hand, it was thought that both HO-1 and BMMSC could exert inhibitory effects and a stronger immunosuppressive effect to better control liver transplant rejection. NK cells are one of the effector cells involved in liver transplant rejection. In the presence of untreated BMMSCs, the activity of NK cells was significantly decreased, but it still showed an upwards trend. After treatment with gene-transfected cells (HO-1 was used), the activity of NK cells was inhibited within the normal range and did not increase significantly. We hypothesize that this occurred because BMMSC prolonged the survival time in vivo and exerted a more sustained immunosuppressive effect. This effect may also be associated with immune regulation by HO-1 overexpression. HO-1 enhanced and prolonged the effects of BMMSC on acute rejection following reduced-size liver transplantation, with immunomodulatory effects in which adaptive and innate immunity, as well as paracrine signalling, may play important roles^29^. However, there are certain limitations in the viability and homing ability of BMMSC. Even after the transplantation of BMMSC in the lesion site, most of the cells die within a few hours. Selecting suitable genes to modify BMMSC may be an effective way to improve efficacy. The analysis of GFP expression in the transfected BMMSC confirmed that HO-1 had been successfully transferred into BMMSC. Adenovirus is often used as the vector for target genes, which has the advantage of high transfection efficiency in dividing and nondividing cells. However, the DNA carried by adenovirus exists freely in the cytoplasm and is not integrated into the genome, which makes the target gene unstable. With passage and the culture of cells, the target gene will be lost, which is a disadvantage of using adenovirus. However, if the cell is stable and not passaged, it can still produce a stable effect of the target gene.

The morphology, surface phenotype and differentiation potential of transgenic BMMSC showed that the transfected BMMSC retained the basic characteristics of normal BMMSC. The transfected genes did not affect the basic characteristics of BMMSC. These results fully confirmed that we successfully obtained BMMSC with basic characteristics through genetic engineering. The efficacy of HO-1/BMMSC was demonstrated in vitro by MLR assays.

In organ transplantation, graft rejection is a T cell-mediated immune response to donor antigens. Th1, Th2, Th17, Treg cells and other CD4^+^ Th cells have different immune effects. The ratios of Th1/Th2 and Th17/Treg cells play important roles in regulating the T cell immune response^30^. Th1 cells mainly secrete IL-2, IFN-γ and TNF-α, while Th2 cells mainly secrete IL-2, IFN-γ, TNF-α, IL-4, IL-10 and IL-13. The Th1/Th2 ratio and related cytokines are often used to explain immune-related phenomena in organ transplantation^31^. Th17 cells are characterized by the secretion of the cytokines IL-17, IL-21 and IL-22. Their differentiation requires the participation of TGF-β and IL-6, and the stabilization of this phenotype requires IL-23, TNF-α and IL-1β^32^. Tregs can produce anti-inflammatory effects and promote self-tolerance by secreting IL-10 and TGF-β. Th17 cells and their cytokines IL-6, IL-17 and IL-23 may mediate transplantation rejection^33^. Tregs may prevent rejection and even induce the maintenance of immune tolerance^34^. The immunoregulation of BMMSC is related to the amplification of Tregs. In some studies, MSC can induce Foxp3^+^ and Treg-dependent tolerance^35^.

In this study, inflammation and Th cell differentiation-related cytokines were significantly changed in response to treatment with BMMSC and HO-1/BMMSC. Specifically, the concentrations of IL-10 and TGF-β increased significantly, while the concentrations of IL-2, IL-6, IL-17, IL-23, TNF-α and IFN-γ decreased significantly. Compared with BMMSC group, HO-1/BMMSC group had increased expression of IL-10 and TGF-β and decreased expression of IL-2, IL-6, IL-17, IL-23, TNF-α and IFN-γ, and the rising trend in IL-6, IL-17 and IL-23 was reversed to some extent. The change in Tregs was also significant. HO-1/BMMSC upregulated Treg expression, which was stable and lasting. We hypothesized that these effects were related to protecting BMMSC and enhancing the immune regulation of HO-1.

In the BN → BN,LEW → LEW model, we assessed for rejection with clinical parameters such as the mental status, activity, diet, water intake, weight, and so on. These results indicated that the syngraft model has no obvious rejection occurred and immune tolerance was achieved finally. Therefore, to obtain transplanted liver by relaparotomy and perform histopathological examination were not necessary so as to avoid waste of experimental resources. Naturally, the Banff as an histopathological evaluation criteria for rejection was not needed to use in the syngraft model. Due to immune tolerance, transfection of BMMSC and BMMSC/HO-1 into the syngeneic transplantation group is of little scientific significance. The syngraft model also revealed from the opposite side that HO-1/BMMSC play a very important role in inhibiting rejection in allotransplantation of the liver.

In summary, compared to that of BMMSC alone, HO-1/BMMSC-mediated inhibition of liver transplant rejection was stronger and lasted longer, possibly due to the interaction between NK cells, Tregs, and relevant cytokines.

### Materials

DMEM/F12 (1:1) (HyClone, USA), foetal bovine serum (PAA, Australia), glutamate, penicillin/streptomycin double-antibody (Gibco, USA), trypsin, dimethyl sulfoxide, phosphate buffer solution (Sigma, USA), 10× red blood cell lysis buffer (BD, USA), rat IL-10, TGF-β, IL-2, IL-17, IL-23, and TNF-α kits (Santa Cruz Biotechnology, USA), recombinant adenovirus expressing rat HO-1 (Shanghai Jikai Gene Chemical Technology Co. Ltd, China). ^3^H-TdR (Shanghai Institute of Atomic Energy), a Leica 0269 surgical microscope (Leica microsystems AG, Germany), microsurgical instruments (Admiralty Medical Devices, China), an upright fluorescence microscope (Nikon Ni-U, Japan), a flow cytometer (BD FACSCalibur, USA), an enzyme standard instrument (Bio Tek Synergy 2, USA), and a Centricon Plus-20 centrifugal ultrafiltration device (Millipore, USA) were used.

### Animal experimental groups

Experimental animals were purchased and maintained in our Experimental Animal Centre under specific pathogen-free conditions. The procedures for the use of rats in this study were approved by the Animal Care and Ethics Committee of Hubei University of Medicine. Healthy male BN rats (RT-1^n^) and male Lewis rats (RT-1^1^) (BN rats weighing 180–200 g and Lewis rats weighing 200–220 g) were used. The animals were placed in an environment with a 12-h light–dark cycle and were fed adaptively for 7 days. BN rats were used as donors, and Lewis rats were used as recipients to establish an allogeneic acute rejection model (BN → LEW). Rats were injected with NS or cells via the dorsal penile vein before the operation. The animals were divided into three groups: NS group, BMMSC group and HO-1/BMMSC group. A total of 70 rats (35 pairs) were operated on in each group. At 0, 1, 5, 7, and 10 days, five rats were randomly selected for study. Survival time, clinical evolutions and body weight were observed for the rest of the rats. The experimental group was administered 5 × 10^6^/ml cells (1 ml/rat). The control group was given NS (1 ml/rat). The rats were euthanized at 10 days after transplantation, and peripheral venous blood and tissue samples were collected. The rats were handled according to the recommendations in the ARRIVE guidelines.

### The establishment of liver transplantation model

Orthotopic liver transplantation was performed according to previous studies^36^. Anaesthesia was induced by an intraperitoneal injection of 5% chloral hydrate in the donor. We incised the abdomen, dissociated the liver peripheral ligament and separated the portal vein and hepatic inferior vena cava. We incised the common bile duct and placed biliary stents into it. We punctured the portal vein to perfuse lactate Ringer's solution. We cut off the suprahepatic and infrahepatic inferior vena cava as outflow. When the liver was a dusty yellow colour, the portal vein and common hepatic artery were cut off, and the donor liver was excised and removed. The tissues around the suprahepatic and infrahepatic inferior vena cava were trimmed. The donor liver was stored at 4 °C in lactate Ringer's solution for transplantation. When the portal vein, suprahepatic and infrahepatic inferior vena cava and proper hepatic artery were removed, the recipient liver was removed. The donor liver was placed. The portal vein and infrahepatic inferior vena cava were anastomosed with a cuff. The suprahepatic inferior vena cava was anastomosed along the diaphragm ring with an end-to-end running suture. The donor bile duct stent was inserted into the recipient common bile duct and fixed. The greater omentum covered the bile duct junction. The donor common hepatic artery was anastomosed to the recipient proper hepatic artery. After checking that the cuff was not twisted and suprahepatic and infrahepatic inferior vena cava had no leakage, the abdominal cavity was flushed and the abdomen was closed. HO-1/BMMSCs and BMMSCs (5 × 10^6^/ml) were infused via the portal vein during the operation. Postoperative care involved feeding in a single cage after surgery, drinking freely after waking and eating freely after 12 h. The cage was cleaned and disinfected regularly, and the dressing was changed to keep the wound clean. The survival state, clinical body weight, liver function, and survival time in each group after transplantation were observed. We observed the American Veterinary Medical Association (AVMA) Guidelines for the Euthanasia of Animals (2020).

### Extraction, culture and HO-1 transfection of BMMSCs and their efficacy

The source of BMMSCs were BN rats. BMMSCs were verified based on our previous research^36^. HO-1/BMMSCs were constructed by incubating third-generation BMMSCs with recombinant adenovirus containing the HO-1 gene. Modification of BMMSCs with the HO-1 gene can improve their tolerance of hypoxia and reoxygenation injury and their survival and proliferative abilities. The adenovirus was packaged and transfected into 293 T cells. The supernatant containing the virus was collected and purified by ultrafiltration using a Centricon Plus-20 centrifugal ultrafiltration device (Millipore, USA). The viral titre was 5 × 10^8^ TU/mL by the hole dilution method. We observed and counted BMMSCs expressing green fluorescence under a fluorescence microscope. We found that the fluorescence expression rate at an MOI of 10 was the best. Therefore, we chose an MOI value of 10 to complete the subsequent experiments. Twenty-five days after liver transplantation, fresh liver tissue was collected and made into frozen slices. The dense distribution of HO-1/BMMSCs was observed under a fluorescence microscope to verify treatment efficacy. The expression of HO-1 in HO-1/BMMSCs was detected by cytochemical staining and a semiquantification method that was performed in a blinded manner. A sterilized cover slip was placed in the bottom of a sterile Petri dish, and 5 ml of adenovirus-loaded HO-1 was added to the stained BMMSC suspension (density 1 × 10^6^/ml) in the culture dish. After the cells were grown and had fused, the cells were rinsed 3 times with PBS and treated with 4% paraformaldehyde for 20 min. Then, 0.5% Triton X-100 was added and incubated for 20 min at room temperature, the cells were rinsed with 3% H_2_O_2_ for 15 min at room temperature and then rinsed with 100 ml/L normal sheep serum blocking solution for 1 h. A rabbit anti-mouse HO-1 antibody (1:100) was added to the slides, and the slides were incubated at 4 °C overnight after being washed. After being rinsed, PE-labelled sheep anti-rabbit immunofluorescence antibody (1:400) was added and incubated in a 37 °C water bath for 1 h in the dark. The red fluorescence was observed under a fluorescence microscope after the samples were rinsed.

### Mixed lymphocyte reaction (MLR) in vitro

Splenic lymphocytes from BN and Lewis rats were isolated, and the final concentration was adjusted to 2 × 10^6^/ml. Mitomycin C was added to BN splenic lymphocytes, which were treated as the stimulus cells. Lewis splenic lymphocytes were treated as the stimulus cells. The cells were treated at 37 °C for 1 h, centrifuged and washed with DMEM. The cell concentration was adjusted to 2 × 10^5^/ml with 10% foetal bovine serum. In the control group, the cells (2 × 10^5^/well) were added to 96-well plates, and stimulus cells (2 × 10^5^/well) from BN rats were added. The ratio of HO-1/BMMSCs or BMMSCs to the responsive cells was 1:20. BMMSCs and HO-1/BMMSCs were added (1 × 10^4^/well) on the basis of the above control group. Each group was examined in replicate wells in which DMEM containing 10% foetal bovine serum was added to an adjusted final volume of 250 µl. The cells were cultured in an incubator at 37 °C and 5% CO_2_ for 5 days. ^3^H-TdR was added at a dose of 1 μCi/well and incubated for 18 h before the culture was terminated, and the cells were collected on fibre filter paper. After being dried, the cells were put into a scintillation solution, and the CPM value was measured in a scintillation counter. The inhibition rate was calculated as follows: (%) = (1 − CPM value of the experimental group/CPM value of the control group) × 100%.

### Postoperative histopathological and liver function examination

Histopathological examination was performed in each group 10 days after the operation. The histopathological changes were observed by optical microscopy. According to the criteria of acute rejection, the degree of rejection at each time point in each group of rats was scored, and statistical analysis was performed. Under an optical microscope, 5 fields were randomly selected to observe the pathological changes in the two groups of animals. The liver transplant rejection evaluation was based on the Banff international standard formulated in 1997. Venous blood was extracted from the inferior vena cava and gently placed into a biochemical anticoagulant tube, allowed to stand for more than 5 min and then centrifuged at 1200 RPM. After centrifugation for 15 min, the supernatant was retained. Liver function, including ALT, AST, and TBil, was examined by an automatic biochemical analyser.

### Detection of NK cell activity in recipient

We extracted splenic lymphocytes from each group of recipients and cocultured them with YAC-1 target cells to calculate the activity of NK cells by measuring the amount of lactate dehydrogenase (LDH) released. Statistical analysis was performed by analysing the differences between groups (P < 0.05).

### Detection of immune-related cytokines and Tregs

Enzyme-linked immunosorbent assay (ELISA) was used to measure the levels of TNF-α, IL-10, TGF-β, IL-2, IL-17, and IL-23 in the serum of each group at each time point. The specific steps were performed according to the kit instructions. To detect splenic Tregs by flow cytometry, recipient rat splenic lymphocytes were isolated and resuspended in PBS to a concentration of 1 × 10^7^/ml, and 0.1 ml of the cells was added to 0.5 μl of anti-CD4 and 0.625 μl of anti-CD25 antibodies and incubated at 4℃ for 30 min in the dark. After being washed with PBS, the cells were incubated with 1 ml overnight at 4 °C in the dark. The cells were washed with PBS, diluted to 0.1 ml with 5 μl of anti-Foxp3 antibody, and incubated at 4 °C for 2 h in the dark. After being washed with PBS, the cells were fixed in 4% paraformaldehyde and analysed. The changes in CD4^+^, CD25^+^, and Foxp3^+^ cells were detected by cytometry. The differences between the groups were statistically analysed.

### To establish LT model of the syngraft

The syngraft model (LEW → LEW,BN → BN)was also established. The main clinical features, such as mental state, activity, diet, water intake, body weight, liver function and average survival, were observed to evaluate rejection.

### Statistical analysis

Dataanalysis and graphproduction were performed by using GraphPad Prism 5.0 software. Statistical analysis was performed by using SPSS20. Data comparisons were performed between the two groups using Student's t test. One-way analysis of variance (one-way ANOVA) was used to examine data from 3 or more groups. P < 0.05 was considered statistically significant.
